# Effect of malting on physicochemical, antioxidant, and microstructural properties of finger millet (*Eleusine coracana*) flours

**DOI:** 10.1002/fsn3.3790

**Published:** 2023-10-31

**Authors:** Kundai Thelma Murungweni, Shonisani Eugenia Ramashia, Mpho Edward Mashau

**Affiliations:** ^1^ Department of Food Science and Technology, Faculty of Science, Engineering and Agriculture University of Venda Thohoyandou South Africa; ^2^ School of Bioengineering and Food Technology, Faculty of Applied Sciences and Biotechnology Shoolini University Solan India

**Keywords:** functional, germination, Millet, polyphenolic compounds, thermal and morphological characteristics

## Abstract

Finger millet (*Eleusine coracana* L. Gaertn.) is a gluten‐free crop with a high amount of fiber, calcium and iron, outstanding malting qualities and a low glycemic index. The study aimed to determine the physicochemical, functional, antioxidant and microstructural properties of malted finger millet (light and dark brown) flours. The two varieties of finger millet grains were germinated for 0, 24, 48 and 72 h and kilned for 8 h. The lightness (L*) values of malted finger millet flours significantly increased, with light brown having the highest L* value of 76.62. The hue angle and total color differences (Δ*E*) of the malted finger millet flours increased significantly (*p* ≤ .05.), and values ranged from 63.43° to 71.20° (light brown) and 2.12° to 4.32° (dark brown), respectively. The moisture, ash, fiber, protein, total phenolic, total flavonoids contents and DPPH activity of both malted finger millet flours significantly increased. On the contrary, the fat, carbohydrate, energy contents and FRAP activity significantly decreased with each malting period of both finger millet flours. Both malted finger millet flours' solubility index, water and oil absorption capacity increased significantly while the packed and loose bulk density decreased. Malting had no significant effect on the viscosity of the cold paste; however, a significant decrease in the viscosity of the cooked paste in both finger millet flours was observed, with values ranging from 285 to 424.00 cP (light brown) and 271.33 to 418.00 cP (dark brown), respectively. Malting resulted in changes in the thermal properties of finger millet flours with an increase in the onset, peak and conclusion temperatures. Fourier‐Transform Infrared Spectra showed that malting slightly changed the peaks of both finger millet flours. Scanning electron microscopy showed that malting altered the microstructural characteristics of finger millet flours. The results showed that malted finger millet flours are promising raw materials for gluten‐free bakery products.

## Graphical abstract

Physicochemical, antioxidant and microstructural properties of finger millet (*Eleusine coracana*) flours were determined. Malting improved the ash, protein, fiber contents and polyphenolic compounds of finger millet flours. Moreover, malting altered the microstructural properties of finger millet flours. Malted finger millet flours are promising raw materials for gluten‐free bakery products.

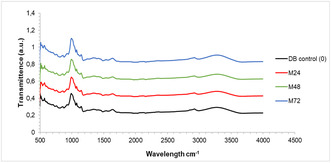

## 1 INTRODUCTION

Finger millet (*Eleusine coracana* L. Gaertn.) is a cereal crop with significant cultural, nutritional and historical importance mainly cultivated in Africa and Asia (Mirza & Marla, 2019; Mueller et al., 2022). The plant's name derives from the crop's panicle shape, forming several fingers‐like structures (Sood et al., 2019). Finger millet is a tiny, seeded cereal crop belonging to the *Poaceae* grass family. The crop has many other names, such as *mufhoho, uphoko* in South Africa, *poho* and *rapoko* in Zimbabwe, *madua* or *ragi* in India and *tokuso* or *dagussa* in Ethiopia (Gull et al., 2014; Ramashia et al., 2019). Finger millet is ranked sixth in production in India among other cereals such as sorghum, rice, wheat, bajra and maize. It is a drought‐resistant crop with high antioxidant and nutraceutical properties (Kandel et al., 2019). It is a gluten‐free cereal crop high in fiber, calcium and iron with outstanding malting qualities and a low glycemic index (Anagha, 2023; Singh, 2016; Udeh et al., 2018). Thus, finger millet is a healthy food choice for diabetic and gluten‐intolerant patients (Patil et al., 2023; Wafula et al., 2018). Millets products are gaining popularity in recent years because of global warming, water shortage, population growth and health concerns of gluten‐containing foods (Aljobair, 2022). Moreover, the health‐promoting compounds of malted cereal grains are attracting high interest as functional ingredients applied to decrease the risk of some chronic diseases such as colorectal cancer (Lin et al., 2019; Nelson et al., 2013).The finger millet grains' flour is utilized as a whole meal to prepare traditional foodstuffs such as pancakes, unleavened bread, dumplings and thin or soft porridge (Sood et al., 2019). Application of flour in food system depends on its functional property (Igbabul et al., 2014; Ojha et al., 2018).

Malting is a food processing technique that has been employed for years to transform and increase the nutritional qualities of millets (Adebiyi et al., 2016; Gowda et al., 2022; Hejazi & Orsat, 2016). Malting practices differ between countries, also vary among communities within a country (Alowo et al., 2018; Bokulich & Bamforth, 2013) and malted cereals are a vital part of the everyday diet of people (Swami et al., 2013; Syeunda et al., 2020). Adetokunboh et al. (2022) stated that malting caused an increment in the activities of hydrolytic enzymes, improving total sugars, amino acids content, B‐group vitamins and a decrease in starch and dry matter. Malting of finger millet grains for brewing and child feeding has been traditionally practiced in many parts of Africa, namely South Africa, Zimbabwe, Nigeria, Kenya, Rwanda and India (Adebiyi et al., 2018; Kubo, 2016). In addition to its nutritional advantages, malting provides an easy, less expensive method of lowering paste viscosity and raising the calorie content of cereal slurries (Baranwal, 2017). The traditional finger millet malting process is similar to sorghum (Embashu & Nantanga, 2019). It includes steeping for 24 h by continuous immersion. Germination is conducted for two to three days, resulting in sprout formation. It is then followed by solar energy dehydration for one to two days at 25–30°C (Adetokunboh et al., 2022).

Functional qualities are a food item's physical and chemical characteristics that influence its behavior during preparation, storage and consumption (Sachdev et al., 2021). Flour is a flexible ingredient that may be utilized in various food items to enhance their nutritional content, sensory and texture quality due to its functional qualities (Awuchi et al., 2019). The cereal‐based finger millet flour ingredient has several valuable qualities, making it a desirable addition to food compositions (Budhwar et al., 2020). These characteristics include the capacity to absorb water and oil, the stability of foam and emulsions, rheological characteristics, gelatinization, flavor and scent (Abah et al., 2020). Water absorption capacity refers to the functional property of a food ingredient or item to take in water when mixed or immersed (Adanse et al., 2021). This property is essential for determining food products' texture, stability and overall quality properties (Abah et al., 2020). Oil absorption capacity is the physical trapping of oil, which retains flavor and enhances food taste for consumers (Abah et al., 2020). The body uses fat for various reasons, a vital part of the diet (Hiremath & Geetha, 2019). The mouthfeel of a food item and the choice of the packaging material can be influenced by the bulk density of the food material (Awuchi et al., 2019). The lower bulk density of the flour improves the digestion of food products, especially in children with undeveloped digestive systems (Ikujenlola & Ogunba, 2018). Differential scanning calorimetry determines the least energy needed to break down a food product's starch structure. Some elements affecting starch are granule shape and concentration (Leyva‐Porras et al., 2019; Shahzad et al., 2019). Finger millet flour is rich in fiber, a vital biological and nutritional element of the human body as it increases the bulk of a diet (Hiremath & Geetha, 2019). Soluble fiber decreases cholesterol and controls blood sugar levels. The insoluble fiber helps in promoting regular bowel motions and preventing constipation (Khalid et al., 2022; Laxmi et al., 2015).

Free radicals are dangerous molecules that can injure cells and tissues but are neutralized by antioxidants which shield the body from oxidative stress (Kabel, 2014; Kumar, Kaur, et al., 2021; Sharma et al., 2015). There are a lot of phenolic compounds and antioxidants in finger millet, and these have several health advantages. The finger millet bran and germ of the grain contain most of these antioxidants (Sharma et al., 2018). Malting also increases finger millet flour's antioxidant activity (Sharma et al., 2022). The flours have a prominent level of antioxidants, making it a beneficial component of functional foods and supplements meant to boost well‐being and fend off chronic diseases (Kumar, Kaur, et al., 2021).

However, there is inadequate research done on the thermal and functional properties of malted finger millet and other millet flours. Nefale and Mashau (2018) observed modifications in the functional characteristics of germinated finger millet flours. Adebiyi et al. (2016), Olamiti et al. (2020), and Mudau et al. (2022) studied modifications in functional and thermal characteristics of malted and fermented finger millet and pearl millet flours. Finger millet flour has good thermal stability and can withstand elevated temperatures without losing its nutritional value or functional properties (Mudau et al., 2022). However, despite the potential benefits of malted finger millet flour, there has been limited research done since it is not widely cultivated compared to other cereal grains such as sorghum, maize and rice. It is important to research malting because it might improve the nutritional value of finger millet flours, improving their worth as a food source in areas where malnutrition is a problem. Malting might contribute to finger millet's grain palatability and digestibility, which might boost its demand. Moreover, it might give farmers and the food industry opportunities to market new value‐added products, boosting revenue and supporting sustainable agricultural methods. Therefore, the study determined the physicochemical, antioxidant and microstructural properties of malted finger millet flours.

## 2 MATERIALS AND METHODS

### 2.1 Materials and reagents

Ten kilograms of light brown (LB) and dark brown (DB) finger millet grains were obtained from street vendors and local markets in Thohoyandou, Limpopo, South Africa. Analytical grade reagents and chemicals were purchased from Merck, Chemicals (PTY) Ltd., Germiston, South Africa.

### 2.2 Malting of finger millet grains and flour production

The LB and DB grains were cleaned with cold tap water to remove foreign materials such as dirt and stones. They were placed in a 5 L cold‐water bucket and left to soak for 10 h at 30°C. After soaking, the grains were drained, weighed and divided into four equal sections. Individual finger millet grains were placed on clean cheese fabrics and permitted to germinate for 0, 24, 48, and 72 h. Water was sprayed onto finger millet grains every 4 h interval to promote germination. The grains were kilned for 8 h and then sprouted in an oven dryer at 50°C, resulting in a distinct malt fragrance. The kilned grains were milled into flours using a miller (Retsch ZM 200, Germany). The samples were stored at 20°C using polyethylene bags for further laboratory analysis. Two different batches of malted finger millet flours were prepared and analyses were duplicated for reliability.

### 2.3 Determination of color properties

The color properties of LB and DB finger millet flours were measured using a ColourFlex spectrophotometer (Hunter Associates Laboratory in Reston, Virginia, USA) after calibration with white and black tiles. The color was interpreted by Hunter values such as L*, a* and b*. The a* designated hue on the green (−) to red (+) axis, whereas b* denoted hue on the blue (−) to yellow (+) axis, yellowness index (YI) denoted yellowness and whiteness index (WI) denoted whiteness. L* denotes lightness, which runs from black to white (0–100). The chroma (C), hue angle (H°), total color difference (*E*), YI and WI were calculated using Equations (1–5).

(1) $$\Delta E=\sqrt{{\left(L*-\mathrm{Lc}\right)}^{2}+{\left(a*-\mathrm{ac}\right)}^{2}+{\left(b*-\mathrm{bc}\right)}^{2}}$$

(2) $$\mathrm{Hue}\;\left(H{}^{\circ}\right)=\tan^{-1}\left\{\left.\frac{b*}{a*}\right\}\right.$$

(3) $$\text{Chroma}=\sqrt{{\left(a*\right)}^{2}+{\left(b*\right)}^{2}}$$

(4) $$\mathrm{YI}=\frac{142.86b*}{L*}$$

(5) $$\mathrm{WI}=\sqrt{{\left(100-L*\right)}^{2}+{\left(a*\right)}^{2}+{\left(b*\right)}^{2}}$$

### 2.4 Proximate composition analysis

Moisture, ash, fat, protein and fiber contents of finger millet flours were determined with the method of AOAC (2016). Method No. 934.01 was used for moisture, 978.10 for protein, 923.03 for ash, 920.39 for fat and 990.03 for fiber contents. The carbohydrate content of malted finger millet flour was determined based on difference method as described by Farzana et al. (2017).

(6) $$\text{Carbohydrate}\;\left(\%\right)=100-(\%\text{Moisture}+\%\text{Protein}+\%\mathrm{Ash}+\%\mathrm{Fat}+\%\text{Fiber}$$

The energy value of malted finger millet flour was determined as stated by Farzana and Mohajan (2022)

(7) $$\text{Energy value}\;\left(\text{kcal}\right)=g/100\;g\;\text{carbohydrate}\times 4+g/100\;g\;\mathrm{fat}\times 9+g/100\;g\;\text{protein}\times 4+g/100\;g\;\text{fiber}\times 2$$

### 2.5 Determination of functional characteristics

#### 2.5.1 Packed and loose bulk density

The density of finger millet flours was calculated using the procedure suggested by Amandikwa et al. (2015), which involves measuring the packed bulk density and loose bulk density. Ten grams of finger millet flour were weighed and placed in a measuring cylinder of 25 mL. The packed bulk density was established by slightly beating the cylinder at the bottom repeatedly until the flour volume became constant. The finger millet flour mass per unit volume was utilized to calculate the packed and loose bulk densities (g/cm^3^).

(8) $$\text{Bulk density}=\frac{\text{Flour weight}\;\left(g\right)}{\text{Volume}\;{\mathrm{cm}}^{3}}$$

#### 2.5.2 Water and oil absorption capacity

Water and oil absorption capacities of finger millet flours were measured using the method described by Mudau et al. (2022). One gram of finger millet flour was weighed into a 50 mL centrifuge tube, and 10 mL of sunflower oil was added. The mixture was mixed thoroughly using a vortex stirrer for 30 min at room temperature (25°C) and centrifuged for 25 min at 3000 rpm (Rotina 380 R‐Labotech Ecotherm centrifuge, Midrand, South Africa). The volume of water or oil absorbed was measured by subtracting the original volume from the volume after centrifugation.

#### 2.5.3 Swelling power

The swelling power of malted finger millet flours was assessed using a method stated by Adebiyi et al. (2016). Finger millet flours were added to a 100 mL measuring cylinder until it reached the 10 mL level and filled with distilled water to the 50 mL mark. The measuring cylinder was firmly closed and inverted for 2 min. After that, the measuring cylinder was flipped and left to stand for 30 min. The volume of the flour samples was then measured.

#### 2.5.4 Solubility index

The solubility index of finger millet flours was measured following a method described by Chandrasekar et al. (2022). Zero point one gram of finger millet flour was mixed with 10 mL of distilled water in a centrifuge tube and shaken at 70°C for 30 min, followed by cooling in cold water for 5 min. Afterwards, the tubes were centrifuged at 1700 rpm for 5 min. The liquid portion was evaporated using a water bath at 100°C until it reached a constant weight. The weight of the flour liquid was then used to calculate the solubility.

#### 2.5.5 Viscosity (cold & hot) pastes

The viscosity of the malted finger millet flours was determined using a Brookfield viscometer (RV model, Brookfield Engineering, Inc., Stoughton, USA) following a procedure outlined by Ramashia et al. (2018). A beaker containing 10 g of each finger millet flours was filled with 90 mL of distilled water, and the mixture was left to hydrate for 30 min. The blend was intermittently stirred until it forms a slurry, and the viscosity of the slurry was quantified while the viscosity of the cold paste was observed. A water bath was used to heat the slurry until it reached its boiling point at 95°C. Viscosity was observed after boiling and cooling of the paste at 30°C.

### 2.6 Polyphenolic compounds and antioxidant activity measurement

Fifty grams of finger millet flour was mixed with 500 mL of methanol. The mixture was then centrifuged (Rotina 380 R‐Labotec Ecotherm (Pty) Ltd, Midrand, SA) for 10 min at 3000 rpm (Mudau et al., 2022). Whatsman no.1 filter paper was used to filter the extract and evaporated; different centrifuge tubes were stored in the refrigerator at 4°C until used.

#### 2.6.1 Total phenolic content (TPC)

The TPC extracted from finger millet flours was evaluated using a method by Dimov et al. (2018) with minor adjustments. Zero point two milliliter of the flour sample was incorporated in test tubes with 2.5 mL of Folin–Ciocalteu that had been diluted five times in 5 mL of distilled water. Seven and half milliliters of 15% sodium carbonate was added to the tubes after 5 min, and the mixture was vortexed (Model 36110740, Separation Scientific, South Africa) and afterwards, it was kept in the dark for 30 min. A spectrophotometer (UV‐1600, Shimadzu Tokyo, Japan) was used to measure the absorbance values at 760 nm. The standard curve was produced using gallic acid, and the outcomes were expressed as mg of gallic acid per gram of the sample flour.

#### 2.6.2 Total flavonoid content (TFC)

A slightly altered version of Mahloko et al. (2019) approach was used to determine the TFC of finger millet flours extract. The extract of the flour samples was mixed with 5% NaNO_2_ (0.3 mL) in a tube, and the mixture was left to react for 5 min before 10% AlCl_3_ (0.6 mL) was added. Distilled water and 2 mL of 1 M NaOH were added after 6 min and vortexed. The absorbance values at 510 nm were measured with a spectrophotometer. The quercetin standard (*R* ^2^ = 0.9992) was used to produce the standard curve, and results were presented in milligrams of quercetin per gram of flour samples (mgCE/g).

#### 2.6.3 DPPH (2,2‐Diphenyl‐1‐pycryl‐hydrazyl) free radical scavenging activity

Following the procedure outlined by Ramashia et al. (2021), DPPH assay of finger millet flour samples (LB & DB) was measured. Two milliliters of each sample and 2 mL of 0.1 mM DPPH were mixed in 95% ethanol. After being stirred up ferociously, the combination was left to sit for 30 min at 25°C under low light. A UV spectrophotometer was used to measure the absorbance of the mixture at 517 nm. The standard curve was produced using a gallic acid solution, and results were expressed as a percentage of the inhibition of DPPH radical activity. The calibration curve equation was *y* = 3.6574*x* + 0.0363; *R* ^2^ was 0.9986.

#### 2.6.4 Ferric reducing antioxidant power (FRAP)

The FRAP assay of finger millet flours was measured following a method described by Lou et al. (2017). A test tube containing 100 mL of the extracted sample from malted FM flours combined with 1 mL of methanol was used. The mixture was thoroughly blended with 2.5 mL of 1% K_3_[Fe (C N)_6_] and 0.2 M phosphate buffer before being centrifuged for 20 min at 5000 rpm (Rotina 380 R‐Labotech Ecotherm, Midrand, South Africa). One milliliter of distilled water and 0.1 mM FeCL_3_ solution were added to the resultant supernatant. Utilizing a spectrophotometer, the transmission density of the mixture was then measured at 700 nm. A bigger absorbance combination suggested a larger reducing power.

### 2.7 Determination of thermal characteristics

The thermal characteristics of finger millet flours were measured using differential scanning calorimetry (DSC 4000, Perkin‐Elmer, Shelton, CT, USA). An empty DSC pan was used as the control, and then 25 mg of finger millet flours was placed in a sealed pan on a weighing balance. Heating of the pan was done from 20 to 130°C at a rating of 10°C per min. Pyris thermal system software linked to DSC was used to record the onset, peak and conclusion temperatures as the gelatinization temperature and enthalpy range of malted and control finger millet flours (Mudau et al., 2022).

### 2.8 Fourier‐Transform infrared spectra analysis

The Nicolet 8700 FTIR spectrometer (Thermo Scientific, Inc., located in Santa Clara, CA, USA) was used to analyze the functional groups of finger millet flours following a method by Adebiyi et al. (2016). The spectral analysis included wavelengths spanning from 400 to 4000 cm^−1^. Zero point five grams of flour was prepared and placed on the instrument for analysis, and the spectra of the flour samples was obtained. The instrument ran 32 scans for each collected spectrum.

### 2.9 Scanning electron microscopy (SEM) analysis

The method described by Gull et al. (2015) was used to determine the microstructure of finger millet flours with slight modifications. Using a gold palladium layer, a coater was employed for coating finger millet flour samples. The samples were placed on a sample holder throughout the coating process. To examine the microstructure of finger millet flours, scanning electron microscopy (Model: JSM 6610‐LV, Chicago, IL, USA) was used. The analysis was performed at a magnification of 1000× and a scale of 20 μm.

### 2.10 Statistical analysis

The experiment was duplicated on different days. All analyses were conducted in triplicates, and mean ± standard deviation (SD) was used to present the results. A one‐way analysis of variance (ANOVA) was employed to analyze the data using SPSS software 26.0 (SPSS Chicago, Illinois, USA). The Duncan multiple range test was used to compare mean values with a significance level of *p* ≤ .05.

## 3 RESULTS AND DISCUSSION

### 3.1 Impact of malting periods on the color properties of light and dark brown finger millet flours

The influence of malting periods on the color properties of finger millet (FM) flours is displayed in Table 1. The lightness (L*) of the LB and DB flour samples ranged from 73.92 to 76.62 and 70.56 to 74.71, respectively. The L* values significantly increased in both FM flours. The color of malted FM flour is often lighter than that of control FM flour (Agrahar‐Murugkar et al., 2015). This is because when FM grains were immersed in water during malting, enzymatic activities took place and broke down complex molecules, including the color pigments of the grain (Adetokunboh et al., 2022; Udeh et al., 2018). Nefale and Mashau (2018) observed increased in L* values of germinated FM flours.

**TABLE 1:** Impact of malting on color profile of malted finger millet flours.

Malting time (h)	L*	a*	b*	Chroma	Hue angle (H°)	Total color different (Δ*E*)	YI	WI
LB finger millet flour								
Control	73.92 ± 0.54^a^	3.81 ± 0.13^b^	7.62 ± 0.26^a^	8.52 ± 0.28^a^	63.43 ± 0.41^a^	–	14.73 ± 0.21^a^	102.41 ± 6.21^a^
M24	75.85 ± 0.50^b^	3.25 ± 0.16^a^	8.29 ± 0.85^b^	8.91^bc^ ± 0.75^ab^	68.59 ± 2.93^b^	2.12 ± 0.84^ab^	15.78 ± 0.18^ab^	108.91 ± 6.18^b^
M48	76.42 ± 0.80^c^	3.20 ± 0.13^a^	8.82 ± 0.14^c^	9.38 ± 0.18^b^	70.05^bc^ ± 0.46^b^	2.84 ± 0.78^b^	16.49 ± 0.15^b^	117.33 ± 5.3^c^
M72	76.62 ± 0.81^d^	3.23 ± 0.05^a^	9.49 ± 0.18^d^	10.02 ± 0.18^c^	71.20 ± 0.08^b^	3.34 ± 0.47^b^	16.45 ± 0.14^b^	129.48 ± 7.23^d^
DB finger millet flour								
Control	70.56 ± 0.99^a^	3.65 ± 0.03^d^	7.56 ± 0.06^d^	8.38 ± 0.03^c^	64.23 ± 0.77^a^	–	15.31 ± 0.19^c^	99.59 ± 3.51^c^
M24	74.16 ± 1.34^b^	2.53 ± 0.01^b^	7.09 ± 0.27^c^	7.53 ± 0.26^b^	70.36 ± 0.71^b^	3.80 ± 1.68^b^	13.66 ± 0.11^b^	85.94 ± 2.98^b^
M48	74.57 ± 0.23^c^	2.49 ± 0.04^a^	6.84 ± 0.15^a^	7.29 ± 0.13^a^	70.00 ± 0.62^b^	4.24 ± 0.83^b^	13.10 ± 0.12^a^	82.08 ± 2.75^a^
M72	74.71 ± 0.71^d^	2.65 ± 0.11^c^	6.91 ± 0.11^b^	7.40 ± 0.14^ab^	69.99 ± 0.50^b^	4.32 ± 1.28^b^	13.21 ± 0.13^a^	82.65 ± 2.80^a^

*Note*: Values are illustrated by average ± standard difference. Different letters in the same line are notably different at *p* < .05.

Abbreviations: Δ*E*, total color difference; a*, redness; b*, yellowness, chroma; DB, dark brown; H°, hue angle; L*, lightness; LB, light brown; M, malted (24, 48, 72 h); WI, whiteness index; YI, yellowness index.

The redness (a*) values of the malted and control FM flours ranged from 3.23 to 3.81 (LB) and 2.53 to 3.65 (DB), and the yellowness (b*) values ranged from 7.62 to 9.49 (LB) and 6.84 to 7.09 (DB), respectively. A significant decrease in a* values was observed in both FM flours. Malted FM flours production involves heating and drying, affecting some pigments. The presence of phenolic compound like tannin in the testa and pericarp of the grain, which was reduced by leaching, could be responsible for the color shift during malting (Devi et al., 2014), particularly in a* values. Tannins undergo polymerization reactions when exposed to oxygen and enzymes (Siwela, 2009). These polymers might have influenced the color changes of the malted FM flours. The b* values significantly increased in LB flours with a decrease in DB flours. The decrease in b* color parameter of malted DB flour might be associated with decreased pigment concentration, such as tannins on the surface of FM flours by water absorption (Table 3). Degradation of tannins during soaking for 10 h, probably due to the diffusion of soluble tannins into the water, might have contributed to color loss (Boon et al., 2010). Moreover, some oxidative enzymes such as polyphenol oxidase and peroxidase were activated during malting, which resulted in browning, thus increasing the b* (yellowness) of LB flour. Yenasew and Urga (2023) observed increased b* values in germinated FM varieties.

The chroma of the FM flours ranged from 8.52 to 10.02 (LB) and 7.29 to 8.38 (DB). A significant increase in the chroma of the LB flours was noted with a decrease in DB flours. Korus et al. (2017) and Olamiti et al. (2020) observed that the higher chroma values with the increase in malting resulted in the liberation of glycones from conjugated glycosides triggered by the stimulation of enzymes or by the synthesis of flavonoids. The high concentration of a* and b* color values in LB flours could have increased chroma, and as the concentration decreased, the color got darker (Alotaibi et al., 2021). The leaching of polyphenols during malting might have contributed to the variations in chroma of the malted FM flours (Radonjić et al., 2020).

Hue angle (H°) is the qualitative aspect of color, typically based on greenish, reddish and other hues (Olamiti et al., 2020). Pathare et al. (2013) and Emery et al. (2021) mentioned that hue values between 0° and 90° correspond to the red hue, whereas values beyond 90° correspond to the yellow hue. The hue angle values for the FM flours samples ranged from 63.43° to 71.20° (LB) and 64.23° to 70.36° (DB), respectively. At 72 h of malting, a higher H° was observed in LB flours in contrast to the lower DB flours. However, the hue angles for both malted FM flours were <90°, indicating reddish‐yellow (Kortei & Akonor, 2015). Variations in values of H° of FM flours could be due to differences in malting periods that produced different protein content (Table 2) and soluble sugars. Azeez et al. (2022) observed an increase in hue angle in germinated brown FM flours.

The extent of the color shift between the malted and control FM samples is indicated by the total color difference (Δ*E*) (Wirkijowska et al., 2020). The change of color ranged from 0.00 to 3.34 (LB) and 0.00 to 4.34 (DB). Both FM flour samples showed no significant difference. Amadou and Moussa (2018) observed similar results in germinated millets.

The whiteness index (WI) and the yellowness index (YI) of FM flours are presented in Table 1. The WI values of LB flours significantly increased (*p* < .05) with values ranging from 102.41 to 129.48. Nevertheless, the WI values of DB flour significantly decreased with an increase in malting time. The decrease in WI of malted DB flour might be due to the natural darker color. Nguyen et al. (2022) observed a decrease in WI in germinated millet flours. The YI values significantly increased in LB flours with an increase in malting ranging from 14.73 to 16.45 whereas the YI in DB flours significantly decreased. The decreasing trends in YI of malted DB flours could be attributed to the lower b* values observed.

### 3.2 Proximate composition of malted light and dark brown finger millet flours

The effect of malting periods on the proximate composition of FM flours is displayed in Table 2. A significant increase in the moisture content with the increase in malting time was observed in both FM flours with values ranging from 10.60% to 11.09% (LB) and 10.51% to 11.09% (DB), respectively. During the malting process, the FM grains absorbed water, which triggered the liberation of enzymes that broke down complex starches and proteins into simpler components that the growing plant could use for energy. Thus, the moisture content of the grain increased. Abioye et al. (2018) mentioned that the increase in the moisture content of malted FM flour was linked to the fact that whole grains absorbed moisture from the soaking water during germination, and more cells within the grains were moistened as the soaking duration increased.

**TABLE 2:** Impact of malting time (h) on the proximate composition of finger millet flours.

Malting time (h)	Moisture (%)	Ash (%)	Fiber (%)	Fat (%)	Protein (%)	Carbohydrate (%)	Energy (kcal/100 g)
LB finger millet flour							
Control	10.60 ± 0.80^a^	1.57 ± 0.37^a^	1.90 ± 0.10^a^	1.72 ± 0.09^c^	10.80 ± 0.50^a^	72.03 ± 0.69^a^	353.00 ± 0.75^a^
M24	10.75 ± 0.81^ab^	1.68 ± 0.08^a^	2.36 ± 0.09^b^	1.59 ± 0.08^bc^	11.20 ± 0.87^b^	71.96 ± 0.54^a^	352.66 ± 2.530^a^
M48	10.93 ± 0.54^bc^	1.80 ± 0.03^b^	2.85 ± 0.11^c^	1.42 ± 0.04^ab^	11.89 ± 0.18^c^	71.56 ± 0.04^a^	351.30 ± 0.99^a^
M72	11.09 ± 0.50^c^	2.27 ± 0.04^c^	3.01 ± 0.02^c^	1.36 ± 0.03^a^	12.20 ± 0.36^c^	71.44 ± 0.29^a^	350.50 ± 0.72^a^
DB finger millet flour							
Control	10.51 ± 0.99^a^	1.46 ± 0.40^a^	1.81 ± 0.11^a^	1.70 ± 0.02^c^	10.75 ± 0.55^a^	72.57 ± 0.47^d^	352.53 ± 1.32^d^
M24	10.92 ± 1.34^b^	1.65 ± 0.04^b^	2.38 ± 0.13^b^	1.58 ± 0.10^bc^	11.48 ± 0.07^b^	71.56 ± 0.31^c^	351.97 ± 0.86^c^
M48	11.01 ± 0.23^c^	1.77 ± 0.06^c^	2.82 ± 0.11^c^	1.40 ± 0.05^ab^	11.85 ± 0.35^c^	71.34 ± 0.59^b^	351.17 ± 0.62^b^
M72	11.09 ± 0.71^d^	2.21 ± 0.10^d^	3.01 ± 0.13 ^cd^	1.35 ± 0.03^a^	12.30 ± 0.36^d^	71.24 ± 0.87^a^	349.25 ± 3.39^a^

*Note*: Values are illustrated by average ± standard difference. Different letters in the same column are notably different (*p* < .05).

Abbreviations: DB, dark brown; LB, light brown; M, malted (24, 48, 72 h).

The ash content of the malted FM flours ranged from 1.57% to 2.27% (LB) and 1.46% to 2.27% (DB). There was a significant increase (*p* < .05) in the ash content of malted LB and DB flours from 24 to 72 h. This was a positive effect as it resulted in more nutrient‐rich flour. Guzmán‐Ortiz et al. (2019) also stated that enzymes break down complex proteins and carbohydrates into simpler parts for the growing plant to use as energy during germination. Thus, the longer germination period activated enzymes, and more minerals were released from the grain. Lande et al. (2017) observed a high ash content in malted FM flours.

Malting increased the total dietary fiber (TDF) of FM flours with values ranging from 1.90% to 3.01% (LB) and 1.81% to 3.01% (DB), respectively. As anticipated, a significant increase (*p* < .05) in the TDF of both FM flours as malting increase was observed. As the malting time increased, the concentration of both soluble and insoluble fiber in the grain increased, leading to a subsequent increment in the fiber content of the resulting flours. Obadina et al. (2017) observed an increase in the TDF of pearl millet flour samples with the length of the malting time.

The fat content of finger millet flours ranged from 1.36% to 1.72% (LB) and 1.35% to 1.70% (DB), respectively. A significant decrease in the fat content of both FM flours was observed as the malting period increased. The decrease in fat content in both malted FM flours was attributed to enzymes hydrolyzing triacylglycerol to release free fatty acids during germination. Moreover, β‐oxidation of free fatty acids took place to produce essential energy to support the growth of seeds (Jan et al., 2017; Saithalavi et al., 2021; Xu et al., 2019). As a result, low fat content was expected throughout germination (Cornejo et al., 2015). Gowda et al. (2022) observed a decrease in the fat content of germinated foxtail millet.

The protein content of FM flours ranged from 10.80% to 12.20% (LB) and 10.75% to 12.30% (DB). There was a significant increase in the protein content of both FM flours with the increase in the malting time. The increase in protein content of the malted FM flours might be due the formation of enzymes or an encompassing change following degradation of other constituents (Ijarotimi & Keshinro, 2011; Owheruo et al., 2019). Moreover, the increase in protein content could be the effect of dry weight loss because of the utilization of some fats and carbohydrates during respiration and the synthesis of some amino acids during germination (Devi et al., 2015; Saithalavi et al., 2021). The protein content of FM flours increased with a longer malting time. This was in line with earlier research findings that crop protein content increased during germination (Abioye et al., 2018). Hejazi and Orsat (2017) and Chauhan (2018) observed a high protein content of germinated FM flour.

The carbohydrate content of FM flours ranged from 71.44% to 72.03% (LB) and 71.24% to 72.57% (DB). No significant difference was observed in the LB flour samples, but a significant decrease was noted in DB flour samples. Malting enhanced the enzymatic degradation of carbohydrates in DB flour into simple sugars by stimulating endogenous enzymes like α‐amylase, improving digestibility because starch was broken down to give energy for the seed growth (Oghbaei & Prakash, 2016; Samtiya et al., 2020). Malting reduced the carbohydrate content of grains, which may benefit individuals with certain health conditions, such as diabetes or obesity (Ojedokun et al., 2020). Gowda et al. (2022) observed low carbohydrate content in malted pearl and kodo millet.

The energy content of FM flours ranged from 350.50 to 353.00 kcal/100 g (LB) and 349.25 to 352.53 kcal/100 g (DB), respectively. A significant decrease was observed in the energy value of both malted FM flours compared to both control FM flours. The germination process caused the breakdown of complex carbohydrates, which reduced carbohydrates in the flour and lowered its energy value (Saithalavi et al., 2021). Kulla et al. (2021) observed a decrease in the energy content of malted pearl millet flour.

### 3.3 Impact of malting on functional characteristics of light and dark brown finger millet flours

Table 3 shows the impact of malting on the functional properties of FM flours. The solubility index of FM flours significantly increased with an increase in malting time from 24 to 72 h compared to the control samples. The values ranged from 1.78% to 3.80% (LB) and 1.79% to 3.90% (DB), respectively. The increase in solubility index might be attributed to the elevated changes that starch molecules undergo that caused them to broke down into simpler sugars, resulting in these sugars having a high solubility index for both malted FM flours (Nefale & Mashau, 2018). The malting process decreased the size of the particles in millet flour, resulting in an increase in the surface area of flour, which enhanced its ability to absorb water and, consequently, increased its solubility index (Panda et al., 2020). Kumar, Rani, et al. (2021) found that as the germination time of FM increased, the water solubility index showed a significant increased.

**TABLE 3:** Functional characteristics of malted light brown and dark brown finger millet flours.

Malting time (h)	SOLB (%)	PBD (g/g)	LBD (g/g)	WAC (g/g)	OAC (g/g)	SP (mL)	Visc cold paste (cP)	Visc cooked paste (cP)
LB finger millet flour								
Control	1.78 ± 0.01^a^	0.81 ± 0.01^b^	0.68 ± 0.01^c^	1.55 ± 0.01^a^	1.31 ± 0.01^a^	10.38 ± 0.04^a^	19.59 ± 0.66^a^	424.00 ± 1.00^d^
M24	2.05 ± 0.06^b^	0.79 ± 0.01^b^	0.64 ± 0.03^c^	1.68 ± 0.01^ab^	1.36 ± 0.01^b^	10.00 ± 0.05^a^	19.52 ± 0.67^a^	376.00 ± 1.00^c^
M48	2.93 ± 0.15^c^	0.75 ± 0.01^a^	0.59 ± 0.01^b^	1.70 ± 0.02^d^	1.42 ± 0.01^c^	9.89 ± 0.01^a^	20.00 ± 0.51^b^	335.67 ± 1.15^b^
M72	3.80 ± 0.10^d^	0.74 ± 0.01^a^	0.54 ± 0.01^a^	1.72 ± 0.02^c^	1.49 ± 0.01^d^	9.88 ± 0.01^a^	20.62 ± 0.77^c^	285.00 ± 5.00^a^
DB finger millet flour								
Control	1.79 ± 0.02^a^	0.82 ± 0.02^c^	0.68 ± 0.01^d^	1.54 ± 0.02^a^	1.32 ± 0.01^a^	10.15 ± 0.06^a^	19.59 ± 0.95^a^	418.00 ± 1.00^d^
M24	2.09 ± 0.11^b^	0.79 ± 0.01^b^	0.63 ± 0.02^c^	1.69 ± 0.02^b^	1.36 ± 0.01^b^	10.00 ± 0.03^a^	20.33 ± 0.79^c^	386.00 ± 1.00^c^
M48	3.03 ± 0.15^c^	0.74 ± 0.01^a^	0.58 ± 0.01^b^	1.73 ± 0.01^c^	1.43 ± 0.02^c^	9.92 ± 0.03^a^	20.08 ± 0.99^b^	321.00 ± 1.00^b^
M72	3.90 ± 0.10^d^	0.73 ± 0.10^a^	0.53 ± 0.01^a^	1.78 ± 0.02^d^	1.48 ± 0.01^d^	9.89 ± 0.01^a^	20.46 ± 0.76^d^	271.33 ± 1.53^a^

*Note*: Values are illustrated by average ± standard difference. Different letters in the same column are notably different (*p* < .05).

Abbreviations: cP, specific heat capacity; DB, dark brown; LB, light brown; LBD, loose bulk density; M, malted (24, 48, 72 h); OAC, oil absorption capacity; PBD, packed bulk density; SOLB, solubility; SP, swelling power; Visc, viscosity (cold and cooked paste); WAC, water absorption capacity.

The packed bulk density of malted and control FM flour samples ranged from 0.74 to 0.81 g/mL (LB) and 0.73 to 0.82 g/mL (DB), respectively. The packed bulk density of both FM flour samples significantly decreased with increase in malting periods. The same trend was also observed for loose bulk density. The decrease in bulk density (packed and loose) might be attributed to the degradation of complex composites, such as proteins and starch, caused by alterations during germination (Ocheme et al., 2015). The broken down of starch during malting decreased starch content resulting in low bulk density (Ojha et al., 2018; Oti & Akobundu, 2008). The changes that took place at the molecular level during malting could have resulted in a reduction of interparticle attraction, thereby reducing the bulk density. Low bulk density flour is suitable for infant formulation and might be useful in various bakery products (Okoye et al., 2010). Adebiyi et al. (2016) and Obadina et al. (2017) observed decrease in bulk density of malted finger and pearl millet flours.

The water absorption capacity (WAC) of malted and control FM flours ranged from 1.55 to 1.72 g/g (LB) and 1.54 to 1.78 g/g (DB). A significant increase in the WAC of both FM flours with an increase in malting periods was observed. The formation of molecules such as soluble sugars, which have a high water‐holding capacity, may have contributed to the improved WAC in both malted FM flours (Nefale & Mashau, 2018; Oskaybaş‐Emlek et al., 2021). Abah et al. (2020) stated that flour that can absorb water may contain a higher proportion of hydrophilic components, such as polysaccharides. Moreover, low WAC observed in control FM flours suggests low water‐binding hydrophilic groups (Adebowale et al., 2005; Mudau et al., 2022). Yenasew and Urga (2023) observed similar increases in WAC of germinated FM flours.

The oil absorption capacity (OAC) of FM flour samples ranged from 1.31 to 1.49 g/g (LB) and 1.32 to 1.48 g/g (DB), respectively. The OAC values of both FM flour samples showed a significant increase with malting time. The increase in OAC observed during malting could be attributed to the breakdown of starch during germination, as hydrolyzed starch has a higher capacity to absorb both water and oil (Horstmann et al., 2017). Ojha et al. (2018) observed an increase in the OAC of malted sorghum flour and suggested that the increased ability of sorghum flour to bind with oil indicated its potential usefulness in food formulations where retaining oil was a significant factor to consider. Thus, the increase of OAC in both malted FM flour samples suggested an increased potential in food formulations. Nazni and Shobana (2016) observed an increase in OAC during the germination of barnyard and foxtail millet samples.

The swelling capacity of FM flour samples ranged from 9.88 to 10.38 g/g (LB) and 9.89 to 10.15 g/g (DB), respectively. The swelling power of both malted FM flours significantly decreased with increase in malting time. Low swelling power in both malted FM flours could be attributed to the alterations in protein content or quality, as some proteins may form complexes with starch molecules and reduced their ability to swell (Wilson et al., 2022). Nefale and Mashau (2018) and Yenasew and Urga (2023) noted a decreased of swelling power in germinated FM flours.

The viscosity of FM flours is presented in Table 3. Finger millet flours' cold paste viscosity values ranged from 19.52 to 20.62 cP (LB) and 19.59 to 20.46 cP (DB), respectively. The hot paste values of FM flours ranged from 285.00 to 424.00 cP (LB) and 271.33 to 418.00 cP (DB), respectively. A significant difference in the cold paste viscosity was observed in both FM flours. Malting increased the cold paste viscosity of flour by altering its composition, specifically by increasing the level of soluble fiber and decreasing the amount of starch (Aswalekar et al., 2021; Claver et al., 2010). This increase in soluble fiber was significant since it formed gels that contributed to the cold paste viscosity of the flour and could hold water effectively (Ahmed et al., 2019; Shand, 2000). A significant reduction in the viscosity of the cooked paste with each malting stage in both FM flours was observed. The reduction was mainly caused by alterations in the composition of flour and enzymatic activity, which decreased its starch content (Atuna et al., 2022). The decreased in viscosity in both malted FM flours showed that the flour was suitable for producing infant foodstuffs (Kaushik et al., 2021). Sharma et al. (2021) observed similar results in germinated kodo millet flours.

### 3.4 Impact of malting period on the polyphenols and antioxidant capacity of light brown and dark brown finger millet flours

Table 4 illustrates the polyphenols and antioxidant activity of malted FM flours. Comparing the malted and control FM flours, TPC increased significantly from 67.74 to 94.22 mg (GAE)/100 g (LB) and 68.57 to 95.40 mg (GAE)/100 g (DB). Enzymes that break down the cell wall became functional during malting and altered the structure of the cell wall of the grain, increasing the content of phenolic chemicals in FM flours (Arya, 2022). The malting process decreased the antinutrients like tannins and phytic acid, which can attach to phenolic compounds and decreased their effectiveness. Consequently, removing these antinutrients increased the overall phenolic content of malted finger millet flours (Yousaf et al., 2021). Azeez et al. (2022) recorded an increased TPC in germinated brown FM flour.

**TABLE 4:** Polyphenols and antioxidant activity of malted light brown and dark brown FM flours.

Malting time (h)	TPC (mg GAE/g)	TFC (mg QE/g)	DPPH (%)	FRAP (mg GAE/g)
LB finger millet flour				
Control	67.74 ± 1.01^a^	7.22 ± 1.05^a^	67.31 ± 1.83^a^	0.93 ± 0.00^a^
M24	71.42 ± 1.26^b^	8.48 ± 0.74^ab^	73.32 ± 1.69^b^	0.87 ± 0.02^b^
M48	81.55 ± 1.64^c^	9.30 ± 0.97^b^	82.52 ± 0.86^c^	1.33 ± 0.10^c^
M72	94.22 ± 3.71^d^	10.18 ± 0.95^b^	90.70 ± 2.29^d^	1.88 ± 0.12^d^
DB finger millet flour				
Control	68.57 ± 0.77^a^	7.28 ± 0.94^a^	76.61 ± 0.87^a^	0.96 ± 0.00^a^
M24	72.53 ± 1.26^b^	8.41 ± 1.05^b^	78.02 ± 4.91^b^	0.90 ± 0.02^a^
M48	83.62 ± 1.40^c^	9.41 ± 0.78^c^	87.43 ± 1.38^c^	1.41 ± 0.17^b^
M72	95.40 ± 4.59^d^	10.21 ± 0.41^d^	95.14 ± 1.59^d^	1.87 ± 0.12^c^

*Note*: Values are illustrated by average ± standard difference. Different letters in the same column are notably different at *p* < .05.

Abbreviations: DB, dark brown; DPPH, 2,2‐Diphenyl‐1‐picrylhydrazyl; FRAP, ferric reducing antioxidant power; LB, light brown; M, malted (24, 48, 72 h); TFC, total flavonoids content; TPC, total phenolic content.

The TFC of light and dark brown FM flours ranged from 7.22 to 10.18 mg QE/g (LB) and 7.28 to 10.21 mg QE/g (DB). A significant increase of TFC in both FM flour samples at each malting period was observed. The increase in TFC at each malting period might be because of the metabolic alterations that took place in the grain and produced secondary metabolites or flavonoids (Kaur & Gill, 2021). Moreover, malting resulted in the liberation of glycones from conjugated glycosides triggered by the stimulation of enzymes or by the alteration or synthesis of flavonoids (Sharma et al., 2022). Sharma et al. (2018) observed that malting at 48 h substantially increased the TFC of foxtail millet flour.

The DPPH values significantly increased in both FM flours, ranging from 67.31% to 90.70% (LB) and 76.61% to 95.14% (DB), respectively. The activity of enzymes, released bound antioxidants and reduction of antinutritional factors during malting contributed to the high values of DPPH in both FM flours. The increase in DPPH values suggested that the antioxidant capacity of both FM flours increased, which positively influences consumers' health (Nkhata et al., 2018). Sharma et al. (2021) observed an increment in DPPH radical scavenging activity with malting time in kodo millet. The FRAP values of FM flour samples ranged from 0.87 to 1.88 mg/g GAE (LB) and 0.90 to 1.87 mg/g GAE (DB), respectively. A significant increase in FRAP with the malting periods in both FM flours from 48 and 72 h was observed. The consumption or transformation of antioxidants by the enzymes generated during the malting process could have contributed to low FRAP assay at 24 h in both malted FM flours (Yang et al., 2021).

In contrast, the increase in FRAP assay at 48 to 72 h in both malted FM flours was likely due to the production of new antioxidants and further generation of metabolites produced during malting, as well as the released of phenolic compounds (Yang et al., 2021). Sharma et al. (2018) observed an increase in FRAP in malted foxtail millet flours. Foods that are high in antioxidants may reduce oxidative stress and decrease the risk of conditions like cardiovascular disease, cancer and neurodegenerative disorders (Pham‐Huy et al., 2008).

### 3.5 Thermal characteristics of malted light and dark brown finger millet flours

The thermal properties of the malted and control FM flours are presented in Table 5. The more extended malting period in both the LB and DB flours increased the onset temperature (*T*_*o*_), peak temperature (*T*_*p*_), and conclusion temperature (*T*_*c*_). A significant difference in the malted and control FM flours in relation to the onset, peak and conclusion temperatures was observed. The FM flour samples malted for 72 h exhibited elevated temperatures required for gelatinization. The peak temperature increased after malting could be due to the build‐up of proteolytic enzymes produced by indigenous microorganisms, which degraded the walls of the grain cell, leading to a greater liberation of starch and larger dimensions of crystalline structures in the sample (Mudau et al., 2022). Malting modified the structure of the macromolecular or configuration of amylose and amylopectin present in flour granules and caused variations in gelatinization temperatures (Su et al., 2020). Sharma et al. (2018) and Li et al. (2020) observed an increase of (*T*_*o*_) and (*T*_*p*_) of germinated millet, sorghum and foxtail millet.

**TABLE 5:** Thermal characteristics of malted light brown and dark brown finger millet flours.

Malting time (h)	*T*_*o*_ (°C)	*T*_*p*_ (°C)	*T*_*c*_ (°C)	*T*_*r*_ (°C)	Δ*H* (J/g)
LB finger millet flour					
Control	76.03 ± 1.15^a^	80.41 ± 0.93^a^	86.04 ± 0.45^a^	3.33 ± 1.56^a^	5.38 ± 0.76^d^
M24	78.22 ± 1.09^b^	82.64 ± 0.81^b^	87.21 ± 0.32^b^	3.08 ± 0.63^a^	5.04 ± 0.68^c^
M48	79.62 ± 1.02^c^	83.26 ± 0.72^c^	89.15 ± 0.28^c^	3.65 ± 1.20^a^	4.80 ± 1.12^b^
M72	82.18 ± 0.95^d^	85.21 ± 0.65^d^	90.26 ± 0.15^d^	3.82 ± 1.42^a^	4.32 ± 0.97^a^
DB finger millet flour					
Control	74.16 ± 1.35^a^	75.91 ± 1.26^a^	80.12 ± 0.95^a^	8.78 ± 1.84^a^	4.65 ± 1.72^c^
M24	75.20 ± 1.24^b^	76.18 ± 1.18^b^	80.83 ± 0.92^b^	8.54 ± 1.22^a^	4.52 ± 1.55^c^
M48	76.07 ± 1.14^c^	76.01 ± 1.13^a^	81.13 ± 0.83^c^	8.88 ± 0.56^a^	4.30 ± 0.99^b^
M72	77.52 ± 1.08^d^	77.70 ± 1.09^c^	82.15 ± 0.78^d^	9.38 ± 0.67^a^	4.15 ± 1.09^a^

*Note*: Values are illustrated by average ± standard difference. Different letters in the same column show notable differences (*p* < .05).

Abbreviations: ∆*H*, gelatinization enthalpy; DB, dark brown; LB, light brown; M, malted (24, 48, 72 h); *T*_*c*_, conclusion temperature; *T*_*o*_, onset temperature; *T*_*p*_, peak temperature.

The differences in gelatinization temperatures, including *T*_*o*_, *T*_*p*_ and *T*_*c*_ values, observed in both malted FM flours might be attributed to inherent alterations in granule size, morphology, distribution of starch and organization of internal starch fractions within the granules (Nagaprabha et al., 2017). The observation of increased gelatinization temperatures in both FM flours at 72 h of malting indicated that greater force was necessary to initiate the starch gelatinization. An increase in gelatinization temperatures observed in malted LB and DB flours might be attributed to the generation of amino acids, which resulted from modifying proteins during germination (Azeez et al., 2022; Gebremariam et al., 2014). Starches that exhibited lower gelatinization temperatures, like those in both control and 24 h malted flours, have superior culinary quality (Tomar et al., 2022; Waters et al., 2006). No significant differences were observed in both malted FM flours in relation to the range of gelatinization temperatures.

### 3.6 Fourier‐Transform infrared spectra (FTIR) of malted light and dark brown finger millet flours

**FIGURE 1:**
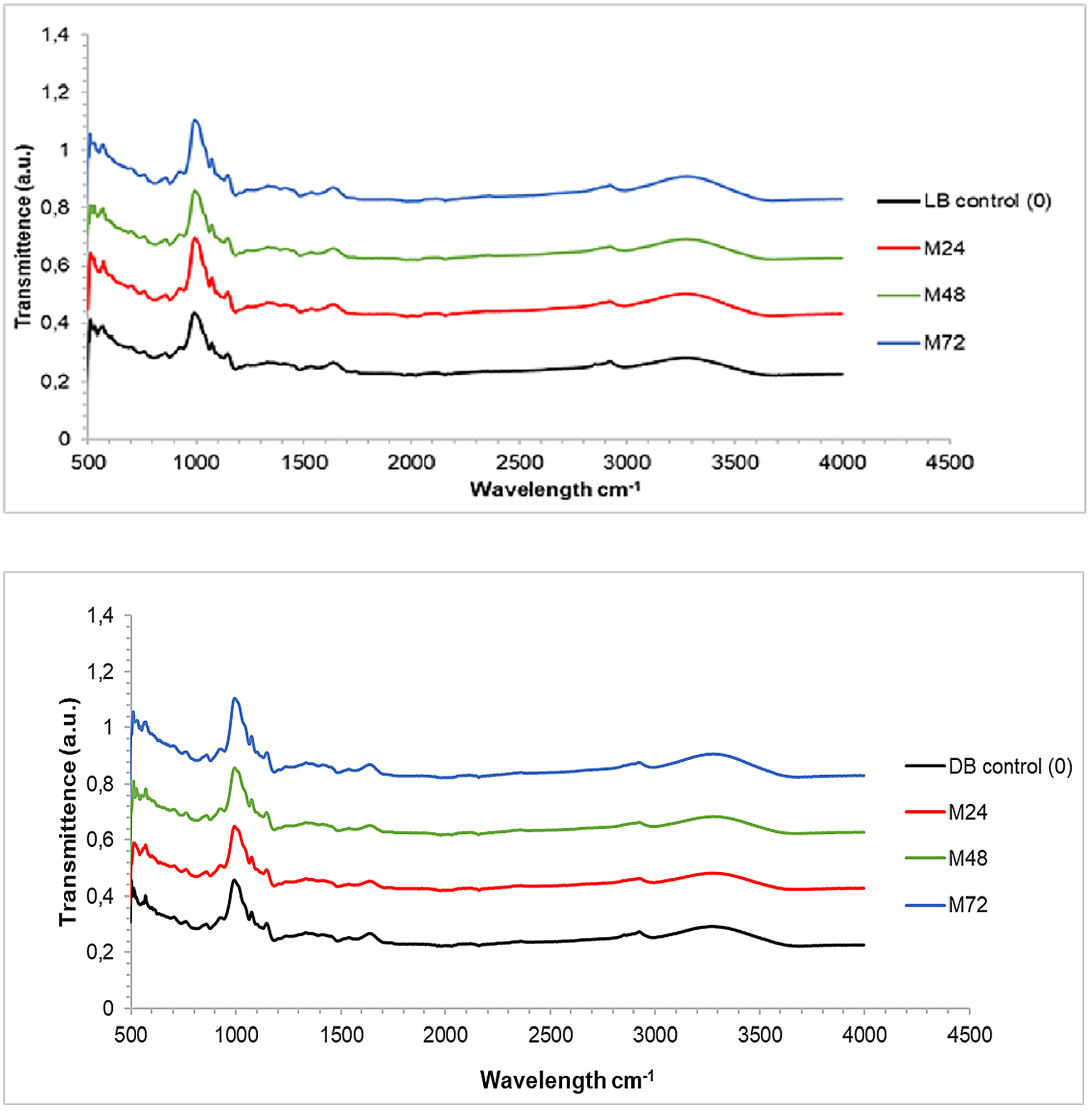
FTIR of malted FM flours. DB control (0), dark brown; FM, finger millet; LB control (0), light brown; M, malted (24, 48, 72 h).

Figure 1 illustrates the FTIR analysis of the functional groups in malted and control FM flours. There were no significant changes in the spectra of malted and control FM flours, but slight variations in the intensity of the peaks were observed. The LB peaks ranged from 3000 to 3600 cm^−1^, and DB peaks from 3000 to 3650 cm^−1^. A broad stretch was observed, indicating the presence of the O‐H group. This was caused by water, alcohol, carboxylic acids, and the interaction between protein and starch (Kaur & Prasad, 2022).

The peaks of the FM flours were in the range of 3253 to 3275 cm^−1^ (LB) and 3252 to 3277 cm^−1^ (DB) in the O‐H region, respectively. The peaks detected in the spectra could be attributed to the vibrations stretch of the O‐H. Variations in the intensity of the peaks might be associated with variations in the moisture content of the flours resulting from the malting process, as shown in Table 2. As the malting period increased, the width of the C‐H band in both malted FM flours widened, with peaks absorption falling between 2914 to 2920 cm^−1^ (LB) and 2925 to 2927 cm^−1^ (DB), respectively. The widening was most likely due to vibrations stretching of both aliphatic and aromatic C‐H bonds (Olamiti et al., 2020). The peak variations could be related to variations in fat content in both malted FM flours (Table 2).

The more pronounced intensities of amide I peak in both malted FM flours might be due to high protein content (Table 2) resulting from the malting of FM flours (Olamiti et al., 2020). Additionally, this study observed many bands in the fingerprint area (800–1600 cm^−1^). FTIR analysis provided important information on the composition of the malted and control FM flours which could be used to analyze quality and nutritional value (Kaur & Prasad, 2022). The peaks shown on the FTIR graph corresponded to various functional groups present in both malted FM flours, and the variations in these peaks provided insights into changes in the flour composition. Adebiyi et al. (2016) observed similar changes in malted pearl millet flour.

### 3.7 Scanning electron morphology of malted light and dark brown finger millet flours

**FIGURE 2:**
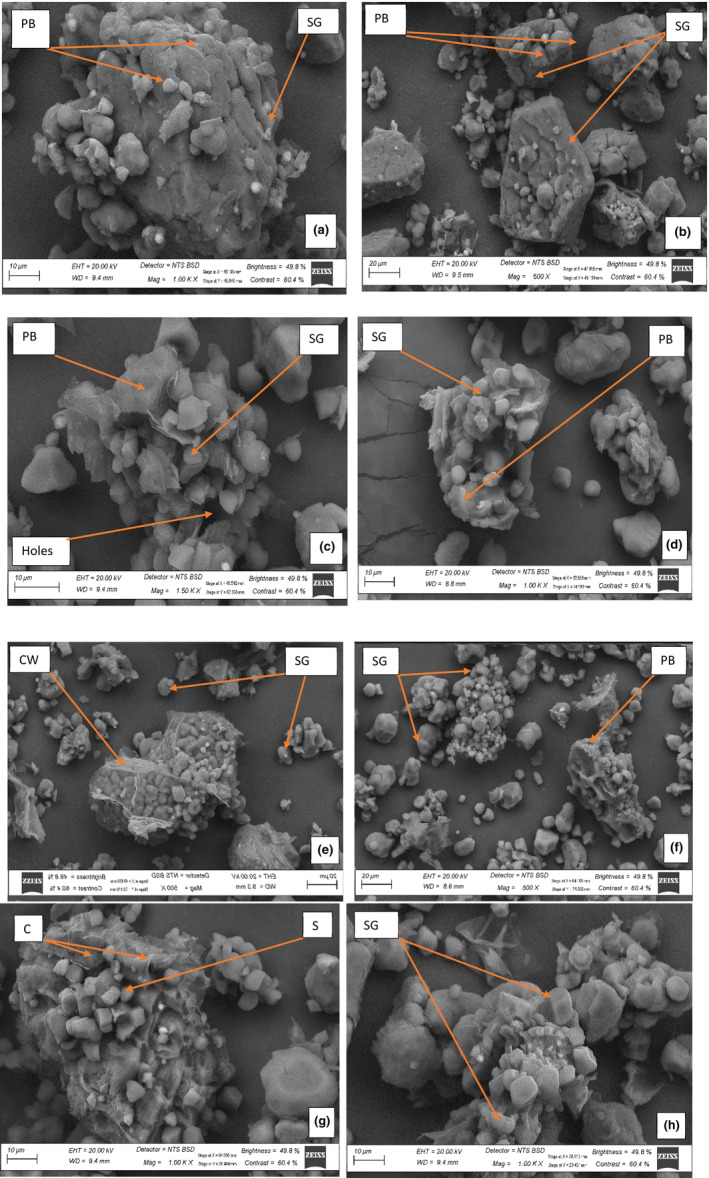
Scanning electron morphology of malted LB and DB finger millet (FM) flours, (a) unmalted LB flours, (b) unmalted DB flours, (c) 24 h malted LB flours, (d) 24 h malted DB flours, (e) 48 h malted LB flours, (f) 48 h malted DB flours, (g) 72 h malted LB flours, (h) 72 h malted DB flours. CW, cell walls; DB, dark brown; FM, finger millet; LB, light brown; PB, protein bodies; SG, starch granules.

Figure 2 illustrates the scanning electron microscopy of malted and control FM flours. The control LB flour had a tightly packed arrangement of protein bodies (PB), but malting caused the breakdown and disintegration of this structure. Both the control LB and DB flours displayed a variety of starch granules (SG) in terms of size, with some being small and others large. Furthermore, the SG was linked together within the PB. On the other hand, the malted FM flours had smaller PB and a greater number of liberated SG than control FM flours. This could be caused by the fact that the process of malting dismantled large compounds of starch into simpler molecules, resulting in the liberation of SG. The liberated SG in both malted FM flours had variations in size and form, ranging from small to large, featuring polygonal shapes, spherical and oval shapes.

Moreover, scanning electron microscopy revealed that both FM malted flours had more holes between the SG than control FM flours. Li et al. (2017) observed pits and holes in germinated starches. Further liberation of SG was observed at 72 h in both malted FM flours, which might have been due to the breakdown of the cell wall. Faltermaier et al. (2015) and Tian et al. (2018) indicated that the cell wall breakdown influenced the SG. The perceived differences in the thermal characteristics, WAC, solubility and swelling power could be the result of the starch composition switching or loosening, as observed in both malted FM flours (Khoza et al., 2021).

## 4 CONCLUSIONS

Variations in nutritional composition in finger millet flours were observed throughout a 72‐h malting. Nutritional composition dictates the physicochemical characteristics as well as food applications. Malting led to a higher mineral content, functional and thermal properties of finger millet flours. The improved thermal stability displayed by both malted finger millet flours renders them suitable for various processing and cooking methods. Malting also modified the microstructural characteristics and functional groups of finger millet flours. Taking into consideration that malting improves the antioxidant properties of finger millet extracts, these malted finger millet flours may be used as functional ingredients in preparing healthy grain‐based products such as weaning foods and gluten‐free bakery products for people suffering from celiac disease.

## AUTHOR CONTRIBUTIONS

**Kundai Thelma Murungweni:** Data curation (equal); formal analysis (equal); investigation (equal); methodology (equal); validation (equal); writing – original draft (lead). **Shonisani Eugenia Ramashia:** Funding acquisition (equal); investigation (equal); project administration (lead); resources (equal); software (equal); supervision (equal); visualization (equal); writing – review and editing (equal). **Mpho Edward Mashau:** Conceptualization (lead); formal analysis (equal); funding acquisition (equal); investigation (equal); supervision (equal); validation (equal); writing – original draft (supporting); writing – review and editing (equal).

## CONFLICT OF INTEREST STATEMENT

All authors declare that there is no conflict of interest in this manuscript.

## Data Availability

The data that support the findings of this study are available on request from the corresponding author.

## References

[fsn33790-bib-0001] Abah, C. R. , Ishiwu, C. N. , Obiegbuna, J. E. , & Oladejo, A. A. (2020). Nutritional composition, functional properties, and food applications of millet grains. Asian Food Science Journal, 14(2), 9–19.

[fsn33790-bib-0002] Abioye, V. F. , Ogunlakin, G. O. , & Taiwo, G. (2018). Effect of germination on antioxidant activity, total phenols, flavonoids, and anti‐nutritional content of finger millet flour. Journal of Food Processing & Technology, 9(2), 1–5.

[fsn33790-bib-0003] Adanse, J. , Bigson, K. , Maureen, N. A. , & Dorothy, A. (2021). Quality characteristics and sensory evaluation of cakes produced from composite blends of wheat (*Titricum aestivum* L.) and finger millet (*Pennisetum glaucum*) flour. Eurasian Journal of Food Science and Technology, 5(2), 190–204.

[fsn33790-bib-0004] Adebiyi, J. A. , Obadina, A. O. , Adebo, O. A. , & Kayitesi, E. (2018). Fermented and malted millet products in Africa: Expedition from traditional/ethnic foods to industrial value‐added products. Critical Reviews in Food Science and Nutrition, 58(3), 463–474. 27246820 10.1080/10408398.2016.1188056

[fsn33790-bib-0005] Adebiyi, J. A. , Obadina, A. O. , Mulaba‐Bafubiandi, A. F. , Adebo, O. A. , & Kayitesi, E. (2016). Effect of fermentation and malting on the microstructure and selected physicochemical properties of pearl millet (*Pennisetum glaucum*) flour and biscuit. Journal of Cereal Science, 70, 132–139.

[fsn33790-bib-0006] Adebowale, Y. A. , Adeyemi, I. A. , & Oshodi, A. A. (2005). Functional and physicochemical properties of flours of six *Mucuna* species. African Journal of Biotechnology, 4(12), 1461–1468.

[fsn33790-bib-0007] Adetokunboh, A. H. , Obilana, A. O. , & Jideani, V. A. (2022). Enzyme and antioxidant activities of malted Bambara groundnut as affected by steeping and sprouting times. Foods, 11(6), 783. 35327205 10.3390/foods11060783 PMC8947651

[fsn33790-bib-0008] Agrahar‐Murugkar, D. , Gulati, P. , Kotwaliwale, N. , & Gupta, C. (2015). Evaluation of nutritional, textural and particle size characteristics of dough and biscuits made from composite flours containing sprouted and malted ingredients. Journal of Food Science and Technology, 52, 5129–5137. 26243934 10.1007/s13197-014-1597-y PMC4519512

[fsn33790-bib-0009] Ahmed, J. , Thomas, L. , & Arfat, Y. A. (2019). Functional, rheological, microstructural and antioxidant properties of quinoa flour in dispersions as influenced by particle size. Food Research International, 116, 302–311. 30716950 10.1016/j.foodres.2018.08.039

[fsn33790-bib-0010] Aljobair, M. O. (2022). Physicochemical properties and sensory attributes of cookies prepared from sorghum and millet composite flour. Food Science and Nutrition, 10, 3415–3423. 36249959 10.1002/fsn3.2942 PMC9548354

[fsn33790-bib-0011] Alotaibi, H. N. , Anderson, A. K. , & Sidhu, J. S. (2021). Influence of lutein content of marigold flowers on functional properties of baked pan bread. Annals of Agricultural Sciences, 66(2), 162–168.

[fsn33790-bib-0012] Alowo, D. , Muggaga, C. , & Ongeng, D. (2018). The effect of traditional malting technology practiced by anethnic community in northern Uganda on in‐vitro nutrient bioavailability and consumer sensory preference for locally formulated complementary food formulae. Food Science and Nutrition, 6, 2491–2498. 30510750 10.1002/fsn3.856 PMC6261209

[fsn33790-bib-0013] Amadou, I. , & Moussa, S. M. A. (2018). Processing and sensory evaluation of germinated millets varieties grains flours. MOJ Food Processing Technology, 6(1), 151–153.

[fsn33790-bib-0014] Amandikwa, C. , Iwe, M. O. , Uzomah, A. , & Olawuni, A. I. (2015). Physico‐chemical properties of wheat‐yam flour composite bread. Nigerian Food Journal, 33, 12–17.

[fsn33790-bib-0015] Anagha, K. K. (2023). Millets: Nutritional importance, health benefits, and bioavailability: A review. Energy, 329(328), 361.

[fsn33790-bib-0016] AOAC . (2016). Official methods of analysis (20th ed.). Association of Official Analytical Chemists.

[fsn33790-bib-0017] Arya, C. (2022). Effect of processing on antioxidant potential and antinutritional factors in small millets. In S. Srivastava (Ed.), Small millet grains. Cereals, pulses and oilseeds (pp. 1–27). Springer Publishing.

[fsn33790-bib-0018] Aswalekar, K. G. , Katke, S. D. , & Pandhare, G. R. (2021). Studies on formulation and quality evaluation of weaning food from sorghum malt. The Pharmacy Innovation Journal, 10(3), 107–113.

[fsn33790-bib-0019] Atuna, R. A. , Ametei, P. N. , Bawa, A. A. , & Amagloh, F. K. (2022). Traditional processing methods reduced phytate in cereal flour, improved nutritional, functional and rheological properties. Scientific African, 15, e01063.

[fsn33790-bib-0020] Awuchi, C. G. , Igwe, V. S. , & Echeta, C. K. (2019). The functional properties of foods and flours. International Journal of Advanced Academic Research, 5(11), 139–160.

[fsn33790-bib-0021] Azeez, S. O. , Chinma, C. E. , Bassey, S. O. , Eze, U. R. , Makinde, A. F. , Sakariyah, A. A. , & Adebo, O. A. (2022). Impact of germination alone or in combination with solid‐state fermentation on the physicochemical, antioxidant, in vitro digestibility, functional and thermal properties of brown finger millet flours. LWT‐Food Science and Technology, 154, 112734.

[fsn33790-bib-0022] Baranwal, D. (2017). Malting: An indigenous technology used for improving the nutritional quality of grains—A review. Asian Journal Dairy Food Research, 36, 179–183.

[fsn33790-bib-0023] Bokulich, N. A. , & Bamforth, C. W. (2013). The microbiology of malting and brewing. Microbiology and Molecular Biology Reviews, 77(2), 157–172. 23699253 10.1128/MMBR.00060-12 PMC3668669

[fsn33790-bib-0024] Boon, C. S. , McClements, D. J. , Weiss, J. , & Decker, E. A. (2010). Factors influencing the chemical stability of carotenoids in foods. Critical Review in Food Science and Nutrition, 50(6), 515–532. 10.1080/10408390802565889 20544442

[fsn33790-bib-0025] Budhwar, S. , Sethi, K. , & Chakraborty, M. (2020). Efficacy of germination and probiotic fermentation on underutilized cereal and millet grains. Food Production, Processing and Nutrition, 2, 1–17.

[fsn33790-bib-0026] Chandrasekar, V. , Nambi, E. V. , Sultan, S. , & John, S. G. (2022). Effect of operating conditions of a solid‐state fermenter on structural and pasting properties of finger millet flour as applied to bread quality. Journal of Food Process Engineering, 45(5), e14017.

[fsn33790-bib-0027] Chauhan, E. S. (2018). Effects of processing (germination and popping) on the nutritional and anti‐nutritional properties of finger millet (*Eleusine coracana*). Current Research in Nutrition and Food Science Journal, 6(2), 566–572.

[fsn33790-bib-0028] Claver, I. P. , Zhang, H. , Li, Q. , Zhu, K. , & Zhou, H. (2010). Impact of the soak and the malt on the physicochemical properties of the sorghum starches. International Journal of Molecular Sciences, 11(8), 3002–3015. 21152287 10.3390/ijms11083002 PMC2996743

[fsn33790-bib-0029] Cornejo, F. , Caceres, P. J. , Martínez‐Villaluenga, C. , Rosell, C. M. , & Frias, J. (2015). Effects of germination on the nutritive value and bioactive compounds of brown rice breads. Food Chemistry, 173, 298–304. 25466026 10.1016/j.foodchem.2014.10.037

[fsn33790-bib-0030] Devi, C. B. , Kushwaha, A. , & Kumar, A. (2015). Sprouting characteristics and associated changes in nutritional composition of cowpea (*Vigna unguiculata*). Journal of Food Science and Technology, 52, 6821–6827. 26396436 10.1007/s13197-015-1832-1 PMC4573095

[fsn33790-bib-0031] Devi, P. B. , Vijayabharathi, R. , Sathyabama, S. , Malleshi, N. G. , & Priyadarisini, V. B. (2014). Health benefits of finger millet (*Eleusine coracana* L.) polyphenols and dietary fibre: A review. Journal of Food Science and Technology, 51, 1021–1040. 24876635 10.1007/s13197-011-0584-9 PMC4033754

[fsn33790-bib-0032] Dimov, I. , Petkova, N. , Nakov, G. , Taneva, I. , Ivanov, I. , & Stamatovska, V. (2018). Improvement of antioxidant potential of wheat flours and breads by addition of medicinal plants. Ukrainian Food Journal, 7, 671–681.

[fsn33790-bib-0033] Embashu, W. , & Nantanga, K. K. M. (2019). Pearl millet grain: A mini‐review of the milling, fermentation and brewing of ontaku, a non‐alcoholic traditional beverage in Namibia. Transactions of the Royal Society of South Africa, 74(3), 276–282.

[fsn33790-bib-0034] Emery, K. J. , Parthasarathy, M. K. , Joyce, D. S. , & Webster, M. A. (2021). Colour perception and compensation in colour deficiencies assessed with hue scaling. Vision Research, 183, 1–15. 33636681 10.1016/j.visres.2021.01.006 PMC8058247

[fsn33790-bib-0035] Faltermaier, A. , Zarnkow, M. , Becker, T. , Gastl, M. , & Arendt, E. K. (2015). Common wheat (*Triticum aestivum* L.): Evaluating microstructural changes during the malting process by using confocal laser scanning microscopy and scanning electron microscopy. European Food Research and Technology, 241, 239–252.

[fsn33790-bib-0036] Farzana, T. , & Mohajan, S. (2022). Effect of incorporation of soy flour to wheat flour on the nutritional and sensory quality of biscuits fortified with mushroom. Food Science and Nutrition, 3(5), 363–369. 10.1002/fsn3.228 PMC4576960 26405522

[fsn33790-bib-0037] Farzana, T. , Mohajan, S. , Saha, T. , Hossain, M. N. , & Haque, M. Z. (2017). Formulation and nutritional evaluation of a healthy vegetable soup powder supplemented with soy flour, mushroom, and moringa leaf. Food Science and Nutrition, 5(4), 911–920. 28748080 10.1002/fsn3.476 PMC5520860

[fsn33790-bib-0038] Gebremariam, M. M. , Zarnkow, M. , & Becker, T. (2014). Teff (*Eragrostis tef*) as a raw material for malting, brewing, and manufacturing of gluten‐free foods and beverages: A review. Journal of Food Science and Technology, 51, 2881–2895. 26396284 10.1007/s13197-012-0745-5 PMC4571201

[fsn33790-bib-0039] Gowda, N. N. , Siliveru, K. , Prasad, P. V. , Bhatt, Y. , Netravati, B. P. , & Gurikar, C. (2022). Modern processing of Indian millets: A perspective on changes in nutritional properties. Food, 11(4), 499. 10.3390/foods11040499 PMC8871339 35205975

[fsn33790-bib-0040] Gull, A. , Jan, R. , Nayik, G. A. , Prasad, K. , & Kumar, P. (2014). Significance of finger millet in nutrition, health, and value‐added products: A review. Magnesium (mg), 130(32), 120.

[fsn33790-bib-0041] Gull, A. , Prasad, K. , & Kumar, P. (2015). Evaluation of functional, antinutritional, pasting and microstructural properties of millet flours. Journal of Food Measurement and Characterization, 10, 96–102.

[fsn33790-bib-0042] Guzmán‐Ortiz, F. A. , Castro‐Rosas, J. , Gómez‐Aldapa, C. A. , Mora‐Escobedo, R. , Rojas‐León, A. , Rodríguez‐Marín, M. L. , Falfán‐Cortés, R. N. , & Román‐Gutiérrez, A. D. (2019). Enzyme activity during germination of different cereals: A review. Food Reviews International, 35(3), 177–200.

[fsn33790-bib-0043] Hejazi, S. N. , & Orsat, V. (2016). Malting process optimization for protein digestibility enhancement in finger millet grain. Journal of Food Science and Technology, 53, 1929–1938. 27413219 10.1007/s13197-016-2188-x PMC4926923

[fsn33790-bib-0044] Hejazi, S. N. , & Orsat, V. (2017). Optimization of the malting process for nutritional improvement of finger millet and amaranth flours in the infant weaning food industry. International Journal of Food Sciences and Nutrition, 68(4), 429–441. 27905218 10.1080/09637486.2016.1261085

[fsn33790-bib-0045] Hiremath, S. P. , & Geetha, K. (2019). Nutritional composition of raw, malted and popped finger millet (Eleusine coracana) varieties. International Journal of Current Microbiology and Applied Sciences, 8(2), 966–974.

[fsn33790-bib-0046] Horstmann, S. W. , Lynch, K. M. , & Arendt, E. K. (2017). Starch characteristics linked to gluten‐free products. Food, 6(4), 29. 10.3390/foods6040029 PMC5409317 28383504

[fsn33790-bib-0047] Igbabul, B. D. , Bello, F. A. , & Ani, E. C. (2014). Effect of fermentation on the proximate composition and functional properties of defatted coconut (*Cocos nucifera L*.) flour sky. Journal of Food Science, 3, 34–40.

[fsn33790-bib-0048] Ijarotimi, O. S. , & Keshinro, O. O. (2011). Determination of amino acid, fatty acid, mineral, functional and choking properties of germinated and fermented popcorn (*Zea mays* everta) flour. European Journal of Food Research and Review, 1, 102–122.

[fsn33790-bib-0049] Ikujenlola, A. V. , & Ogunba, O. B. (2018). Potential complementary food from quality protein maize (*Zea mays*) supplemented with sesame (*Sesamum indicum*) and mushroom (*Oudemansiella radicata*). Journal of Nutrition and Food Science, 8(3), 1–7.

[fsn33790-bib-0050] Jan, R. , Saxena, D. C. , & Singh, S. (2017). Physico‐chemical, textural, sensory and antioxidant characteristics of gluten free cookies made from raw and germinated chenopodium (*Chenopodium album*) flour. LWT‐Food Science and Technology, 71, 281–287.

[fsn33790-bib-0051] Kabel, A. M. (2014). Free radicals and antioxidants: Role of enzymes and nutrition. World Journal of Nutrition and Health, 2(3), 35–38.

[fsn33790-bib-0052] Kandel, M. , Dhami, N. , & Shrestha, J. (2019). Phenotypic diversity of finger millet (*Eleusine coracana* (L.) *Gaertn*.) genotypes. Malaysian Journal of Sustainable Agriculture, 3(2), 20–26.

[fsn33790-bib-0053] Kaur, H. , & Gill, B. S. (2021). Changes in physicochemical, nutritional characteristics and ATR–FTIR molecular interactions of cereal grains during germination. Journal of Food Science and Technology, 58(6), 2313–2324. 33967328 10.1007/s13197-020-04742-6 PMC8076351

[fsn33790-bib-0054] Kaur, R. , & Prasad, K. (2022). Elucidation of chickpea hydration, effect of soaking temperature, and extent of germination on characteristics of malted flour. Journal of Food Science, 87(5), 2197–2210. 35411599 10.1111/1750-3841.16147

[fsn33790-bib-0055] Kaushik, N. , Yadav, P. , Khandal, R. K. , & Aggarwal, M. (2021). Review of ways to enhance the nutritional properties of millets for their value‐addition. Journal of Food Processing and Preservation, 45(6), e15550.

[fsn33790-bib-0056] Khalid, W. , Arshad, M. S. , Jabeen, A. , Muhammad Anjum, F. , Qaisrani, T. B. , & Suleria, H. A. R. (2022). Fiber‐enriched botanicals: A therapeutic tool against certain metabolic ailments. Food Science & Nutrition, 10(10), 3203–3218. 36249968 10.1002/fsn3.2920 PMC9548355

[fsn33790-bib-0057] Khoza, M. , Kayitesi, E. , & Dlamini, B. C. (2021). Physicochemical characteristics, microstructure and health promoting properties of green banana. Food, 10(12), 2894. 10.3390/foods10122894 PMC8700615 34945445

[fsn33790-bib-0058] Kortei, N. K. , & Akonor, P. T. (2015). Correlation between hue‐angle and colour lightness of gamma irradiated mushrooms. Annals in Food Science and Technology, 16, 98–103.

[fsn33790-bib-0059] Korus, A. , Gumul, D. , Krystyjan, M. , Juszczak, L. , & Korus, J. (2017). Evaluation of the quality, nutritional value and antioxidant activity of gluten‐free biscuits made from corn‐acorn flour or corn‐hemp flour composites. European Food Research and Technology, 243, 1429–1438.

[fsn33790-bib-0060] Kubo, R. (2016). The reason for the preferential use of finger millet (*Eleusine coracana*) in eastern African brewing. Journal of the Institute of Brewing, 122(1), 175–180.

[fsn33790-bib-0061] Kulla, S. , Hymavathi, T. V. , Kumari, B. A. , Reddy, R. G. , & Rani, C. V. D. (2021). Impact of germination on the nutritional, antioxidant and antinutrient characteristics of selected minor millet flours. Annals of Phytomedicine International Journal, 10, 178–184.

[fsn33790-bib-0062] Kumar, A. , Kaur, A. , Gupta, K. , Gat, Y. , & Kumar, V. (2021). Assessment of germination time of finger millet for value addition in functional foods. Current Science, 120(2), 406.

[fsn33790-bib-0063] Kumar, A. , Rani, M. , Mani, S. , Shah, P. , Singh, D. B. , Kudapa, H. , & Varshney, R. K. (2021). Nutritional significance and antioxidant‐mediated antiaging effects of finger millet: Molecular insights and prospects. Frontiers in Sustainable Food Systems, 5, 336.

[fsn33790-bib-0064] Lande, S. B. , Thorats, S. , & Kulthe, A. A. (2017). Production of nutrient rich vermicelli with malted finger millet (Ragi) flour. International Journal of Current Microbiology and Applied Sciences, 6(4), 702–710.

[fsn33790-bib-0065] Laxmi, G. , Chaturvedi, N. , & Richa, S. (2015). The impact of malting on nutritional composition of foxtail millet, wheat, and chickpea. Journal of Nutrition and Food Sciences, 5(5), 1–3.

[fsn33790-bib-0066] Leyva‐Porras, C. , Cruz‐Alcantar, P. , Espinosa‐Solís, V. , Martínez‐Guerra, E. , Piñón‐Balderrama, C. I. , Compean Martínez, I. , & Saavedra‐Leos, M. Z. (2019). Application of differential scanning calorimetry (DSC) and modulated differential scanning calorimetry (MDSC) in food and drug industries. Polymers, 12(1), 5. 31861423 10.3390/polym12010005 PMC7023573

[fsn33790-bib-0067] Li, C. , Jeong, D. , Lee, J. H. , & Chung, H. J. (2020). Influence of germination on physicochemical properties of flours from brown rice, oat, sorghum, and millet. Food Science and Biotechnology, 29, 1223–1231. 32802561 10.1007/s10068-020-00770-2 PMC7406585

[fsn33790-bib-0068] Li, C. , Oh, S. G. , Lee, D. H. , Baik, H. W. , & Chung, H. J. (2017). Effect of germination on the structures and physicochemical properties of starches from brown rice, oat, sorghum, and millet. International Journal of Biological Macromolecules, 105, 931–939. 28743574 10.1016/j.ijbiomac.2017.07.123

[fsn33790-bib-0069] Lin, P. Y. , Li, S. C. , Lin, H. P. , & Shih, C. K. (2019). Germinated brown rice combined with *Lactobacillus acidophilus* and *Bifidobacterium animalis* subsp. lactis inhibits colorectal carcinogenesis in rats. Food Science and Nutrition, 7, 216–224. 30680175 10.1002/fsn3.864 PMC6341155

[fsn33790-bib-0070] Lou, Z. , Chen, J. , Yu, F. , Wang, H. , Kou, X. , Ma, C. , & Zhu, S. (2017). The antioxidant, antibacterial, antibiofilm activity of essential oil from *Citrus medica* L. var. sarcodactylis and its nanoemulsion. LWT‐Food Science and Technology, 80, 371–377.

[fsn33790-bib-0071] Mahloko, L. M. , Silungwe, H. , Mashau, M. E. , & Kgatla, T. E. (2019). Bioactive compounds, antioxidant activity and physical characteristics of wheat‐prickly pear and banana biscuits. Heliyon, 5(10), e02479. 31667373 10.1016/j.heliyon.2019.e02479 PMC6812186

[fsn33790-bib-0072] Mirza, N. , & Marla, S. S. (2019). Finger millet (*Eleusine coracana* L. Gartn.) breeding. Advances in Plant Breeding Strategies: Cereals, 5, 83–132.

[fsn33790-bib-0073] Mudau, M. , Ramashia, S. E. , & Mashau, M. E. (2022). Mineral content, functional, thermo‐pasting, and microstructural properties of spontaneously fermented finger millet flours. Food, 11(16), 2474. 10.3390/foods11162474 PMC9407397 36010473

[fsn33790-bib-0074] Mueller, N. G. , Goldstein, S. T. , Odeny, D. , & Boivin, N. (2022). Variability and preservation biases in the archaeobotanical record of *Eleusine coracana* (finger millet): Evidence from iron age Kenya. Vegetation History and Archaeobotany, 31(3), 279–290.

[fsn33790-bib-0075] Nagaprabha, P. , Devisetti, R. , & Bhattacharya, S. (2017). Physicochemical and microstructural characterisation of green gram and foxtail millet starch gels. Journal of Food Science and Technology, 55, 782–791. 29391644 10.1007/s13197-017-2991-z PMC5785405

[fsn33790-bib-0076] Nazni, P. , & Shobana, D. R. (2016). Effect of processing on the characteristics changes in barnyard and foxtail millet. Journal of Food Processing and Technology, 7(3), 1–9.

[fsn33790-bib-0077] Nefale, F. E. , & Mashau, M. E. (2018). Effect of germination period on the physicochemical, functional, and sensory properties of finger millet flour and porridge. Asian Journal of Applied Sciences, 6(5), 360–367.

[fsn33790-bib-0078] Nelson, K. , Stojanovska, L. , Vasiljevic, T. , & Mathai, M. (2013). Germinated grains: A superior whole grain functional food? Canadian Journal of Physiology and Pharmacology, 91(6), 429–441. 23746040 10.1139/cjpp-2012-0351

[fsn33790-bib-0079] Nguyen, T. H. , Vu, D. C. , Ho, T. H. , Nguyet, N. T. , Tuan, N. N. , Thang, T. D. , Trinh, N. T. N. , & Rose, D. J. (2022). Changes in enzymatic activity and in vitro protein digestibility of four millet varieties upon germination and quality evaluation of cookies prepared from germinated millet composite flours. Journal of Food Processing and Preservation, 46(10), e16854.

[fsn33790-bib-0080] Nkhata, S. G. , Ayua, E. , Kamau, E. H. , & Shingiro, J. B. (2018). Fermentation and germination improve nutritional value of cereals and legumes through activation of endogenous enzymes. Food Science and Nutrition, 6(8), 2446–2458. 30510746 10.1002/fsn3.846 PMC6261201

[fsn33790-bib-0081] Obadina, A. O. , Arogbokun, C. A. , Soares, A. O. , de Carvalho, C. W. P. , Barboza, H. T. , & Adekoya, I. O. (2017). Changes in nutritional and physico‐chemical properties of pearl millet (*Pennisetum glaucum*) ex‐Borno variety flour as a result of malting. Journal of Food Science and Technology, 54, 4442–4451. 29184251 10.1007/s13197-017-2922-z PMC5686025

[fsn33790-bib-0082] Ocheme, O. B. , Adedeji, O. E. , Lawal, G. , & Zakari, U. M. (2015). Effect of germination on functional properties and degree of starch gelatinization of sorghum flour. Journal of Food Research, 4(2), 159.

[fsn33790-bib-0083] Oghbaei, M. , & Prakash, J. (2016). Effect of primary processing of cereals and legumes on its nutritional quality: A comprehensive review. Cogent Food and Agriculture, 2, 1136015.

[fsn33790-bib-0084] Ojedokun, F. O. , Ikujenlola, A. V. , & Abiose, S. H. (2020). Nutritional evaluation, glycaemic index and sensory property of breakfast cereals developed from malted amaranth and roasted sesame blends. Scientific Journal of Food Science and Nutrition, 6, 12–19.

[fsn33790-bib-0085] Ojha, P. , Adhikari, R. , Karki, R. , Mishra, A. , Subedi, U. , & Karki, T. B. (2018). Malting and fermentation effects on antinutritional components and functional characteristics of sorghum flour. Food Science and Nutrition, 6(1), 47–53. 29387360 10.1002/fsn3.525 PMC5778236

[fsn33790-bib-0086] Okoye, J. I. , Ezigbo, V. O. , & Animalu, I. L. (2010). Development and quality evaluation of weaning foods fortified with African yam bean flour. Continental Journal Agricultural Science, 4, 1–6.

[fsn33790-bib-0087] Olamiti, G. , Takalani, T. K. , Beswa, D. , & Jideani, A. I. O. (2020). Effect of malting and fermentation on colour, thermal properties, functional groups, and crystallinity level of flours from pearl millet (*Pennisetum glaucum*) and sorghum (*Sorghum bicolor*). Heliyon, 6(12), e05467. 33319084 10.1016/j.heliyon.2020.e05467 PMC7725746

[fsn33790-bib-0088] Oskaybaş‐Emlek, B. , Özbey, A. , & Kahraman, K. (2021). Effects of germination on the physicochemical and nutritional characteristics of lentil and its utilization potential in cookie‐making. Journal of Food Measurement and Characterization, 15(5), 4245–4255.

[fsn33790-bib-0089] Oti, E. , & Akobundu, E. N. T. (2008). Potentials of cocoyam‐soybean‐crayfish mixtures in complementary feeding. Nigeria Agricultural Journal, 39(1&2), 137–145.

[fsn33790-bib-0090] Owheruo, J. O. , Ifesan, B. O. T. , & Kolawole, A. O. (2019). Physicochemical properties of malted finger millet (*Eleusine coracana*) and pearl millet (*Pennisetum glaucum*). Food Science and Nutrition, 7, 476–482. 30847125 10.1002/fsn3.816 PMC6392857

[fsn33790-bib-0091] Panda, D. , Sailaja, N. H. , Padhan, B. , & Lenka, K. (2020). Sprouting‐associated changes in nutritional and physico‐functional properties of indigenous millets from Koraput, India. Proceedings of the National Academy of Sciences, India Section B: Biological Sciences, 90, 79–86.

[fsn33790-bib-0092] Pathare, P. B. , Opara, U. L. , & Al‐Said, F. A. J. (2013). Colour measurement and analysis in fresh and processed foods: A review. Food and Bioprocess Technology, 6, 36–60.

[fsn33790-bib-0093] Patil, P. , Singh, S. P. , & Patel, P. (2023). Functional properties and health benefits of finger millet (*Eleusine coracana* L.): A review. The Journal of Phytopharmacology, 12(3), 196–202.

[fsn33790-bib-0094] Pham‐Huy, L. A. , He, H. , & Pham‐Huy, C. (2008). Free radicals, antioxidants in disease and health. International Journal of Biomedical Sciences, 4(2), 89. PMC3614697 23675073

[fsn33790-bib-0095] Radonjić, S. , Maraš, V. , Raičević, J. , & Košmerl, T. (2020). Wine or beer? Comparison, changes, and improvement of polyphenolic compounds during technological phases. Molecules, 25, 4960. 33120907 10.3390/molecules25214960 PMC7663142

[fsn33790-bib-0096] Ramashia, S. E. , Anyasi, T. A. , Gwata, E. T. , Meddows‐Taylor, S. , & Jideani, A. I. O. (2019). Processing, nutritional composition and health benefits of finger millet in sub‐Saharan Africa. Food Science and Technology., 39(2), 253–266.

[fsn33790-bib-0097] Ramashia, S. E. , Gwata, E. T. , Meddows‐Taylor, S. , Anyasi, T. A. , & Jideani, A. I. O. (2018). Some physical and functional properties of finger millet (*Eleusine coracana*) obtained in sub‐Saharan Africa. Food Research International, 104, 110–118. 29433775 10.1016/j.foodres.2017.09.065

[fsn33790-bib-0098] Ramashia, S. E. , Mamadisa, F. M. , & Mashau, M. E. (2021). Effect of *Parinari curatellifolia* peel flour on the nutritional, physical and antioxidant properties of biscuits. Pro, 9(8), 1262.

[fsn33790-bib-0099] Sachdev, N. , Goomer, S. , & Singh, L. R. (2021). Foxtail millet: A potential crop to meet future demand scenario for alternative sustainable protein. Journal of the Science of Food and Agriculture, 101(3), 831–842. 32767555 10.1002/jsfa.10716

[fsn33790-bib-0100] Saithalavi, K. M. , Bhasin, A. , & Yaqoob, M. (2021). Impact of sprouting on physicochemical and nutritional properties of sorghum: A review. Journal of Food Measurement and Characterization, 15(5), 4190–4204.

[fsn33790-bib-0101] Samtiya, M. , Aluko, R. E. , & Dhewa, T. (2020). Plant food anti‐nutritional factors and their reduction strategies: An overview. Food Production, Processing and Nutrition, 2, 1–14.

[fsn33790-bib-0102] Shahzad, S. A. , Hussain, S. , Mohamed, A. A. , Alamri, M. S. , Ibraheem, M. A. , & Qasem, A. A. A. (2019). Effect of hydrocolloid gums on the pasting, thermal, rheological, and textural properties of chickpea starch. Food, 8(12), 687. 10.3390/foods8120687 PMC6963556 31888161

[fsn33790-bib-0103] Shand, P. J. (2000). Textural, water holding, and sensory properties of low‐fat pork bologna with normal or waxy starch hull‐less barley. Journal of Food Science, 65(1), 101–107.

[fsn33790-bib-0104] Sharma, N. , Goyal, S. K. , Alam, T. , Fatma, S. , Chaoruangrit, A. , & Niranjan, K. (2018). Effect of high pressure soaking on water absorption, gelatinization, and biochemical properties of germinated and non‐germinated foxtail millet grains. Journal of Cereal Science, 83, 162–170.

[fsn33790-bib-0105] Sharma, R. , Bhandari, M. , Sharma, S. , & Bhardwaj, R. (2022). Compositional, structural, and functional characteristics of millets as modified by bioprocessing techniques: A review. Journal of Food Processing and Preservation, 46(10), e16885.

[fsn33790-bib-0106] Sharma, S. , Jan, R. , & Riar, C. S. (2021). Analysing the effect of germination on the pasting, rheological, morphological, and in‐vitro antioxidant characteristics of kodo millet flour and extracts. Food Chemistry, 361, 130073. 34029901 10.1016/j.foodchem.2021.130073

[fsn33790-bib-0107] Sharma, S. , Saxena, D. C. , & Riar, C. S. (2015). Antioxidant activity, total phenolics, flavonoids and antinutritional characteristics of germinated foxtail millet (*Setaria italica*). Cogent Food & Agriculture, 1(1), 1081728.

[fsn33790-bib-0108] Singh, E. (2016). Potential functional implications of finger millet (*Eleusine coracana*) in nutritional benefits, processing, health, and diseases: A review. International Journal of Home Science, 2(21), 151–155.

[fsn33790-bib-0109] Siwela, M. (2009). Finger millet grain phenolics and their impact on malt and cookie quality. Doctoral dissertation. University of Pretoria.

[fsn33790-bib-0110] Sood, S. , Joshi, D. C. , Chandra, A. K. , & Kumar, A. (2019). Phenomics and genomics of finger millet: Status and prospects. Planta, 250, 731–751. 30968267 10.1007/s00425-019-03159-6

[fsn33790-bib-0111] Su, C. , Saleh, A. S. , Zhang, B. , Feng, D. , Zhao, J. , Guo, Y. , Zhao, J. , Li, W. , & Yan, W. (2020). Effects of germination followed by hot air and infrared drying on properties of naked barley flour and starch. International Journal of Biological Macromolecules, 165, 2060–2070. 33096180 10.1016/j.ijbiomac.2020.10.114

[fsn33790-bib-0112] Swami, S. B. , Thakor, N. J. , & Gurav, H. S. (2013). Effect of soaking and malting on finger millet (*Eleusine coracana*) grain. Agricultural Engineering International: CIGR Journal, 15(1), 194–200.

[fsn33790-bib-0113] Syeunda, C. O. , Anyango, J. O. , Faraj, A. K. , & Kimurto, P. K. (2020). In vitro protein digestibility of finger millet complementary porridge as affected by compositing precooked cowpea with improved malted finger millet. Journal of Food Science and Technology, 58, 571–580. 33568850 10.1007/s13197-020-04569-1 PMC7847920

[fsn33790-bib-0114] Tian, J. , Chen, S. , Zhang, H. , Fang, H. , Sun, Y. , Liu, D. , Linhart, R. J. , & Ye, X. (2018). Existing cell wall fragments modify the thermal properties and hydrolysis of potato starch. Food Hydrocolloids, 85, 229–232.

[fsn33790-bib-0115] Tomar, M. , Bhardwaj, R. , Verma, R. , Singh, S. P. , Krishnan, V. , Kansal, R. , Yadav, V. K. , Praveen, S. , & Sachdev, A. (2022). Interactome of millet‐based food matrices: A review. Food Chemistry, 385, 132636. 35339804 10.1016/j.foodchem.2022.132636

[fsn33790-bib-0116] Udeh, H. O. , Duodu, K. G. , & Jideani, A. I. O. (2018). Effect of malting period on physicochemical properties, minerals, and phytic acid of finger millet (*Eleusine coracana*) flour varieties. Food Science and Nutrition, 6(7), 1858–1869. 30349675 10.1002/fsn3.696 PMC6189621

[fsn33790-bib-0117] Wafula, W. N. , Korir, N. K. , Ojulong, H. F. , Siambi, M. , & Gweyi‐Onyango, J. P. (2018). Protein, calcium, zinc, and iron contents of finger millet grain response to varietal differences and phosphorus application in Kenya. Agronomy, 8(2), 24.

[fsn33790-bib-0118] Waters, D. L. , Henry, R. J. , Reinke, R. F. , & Fitzgerald, M. A. (2006). Gelatinization temperature of rice explained by polymorphisms in starch synthase. Plant Biotechnology Journal, 4, 115–122. 17177790 10.1111/j.1467-7652.2005.00162.x

[fsn33790-bib-0119] Wilson, A. , Elumalai, A. , Moses, J. A. , & Anandharamakrishnan, C. (2022). Effect of processing on functional characteristics, physiochemical properties, and nutritional accessibility of millets. In Handbook of millets‐processing, quality, and nutrition status (pp. 205–229). Springer Nature.

[fsn33790-bib-0120] Wirkijowska, A. , Zarzycki, P. , Sobota, A. , Nawrocka, A. , Blicharz‐Kania, A. , & Andrejko, D. (2020). The possibility of using by‐products from the flaxseed industry for functional bread production. LWT‐Food Science and Technology, 118, 108860.

[fsn33790-bib-0121] Xu, M. , Jin, Z. , Simsek, S. , Hall, C. , Rao, J. , & Chen, B. (2019). Effect of germination on the chemical composition, thermal, pasting, and moisture sorption properties of flours from chickpea, lentil, and yellow pea. Food Chemistry, 295, 579–587. 31174798 10.1016/j.foodchem.2019.05.167

[fsn33790-bib-0122] Yang, B. , Yin, Y. , Liu, C. , Zhao, Z. , & Guo, M. (2021). Effect of germination time on the compositional, functional and antioxidant properties of whole wheat malt and its end‐use evaluation in cookie‐making. Food Chemistry, 349, 129125. 33535111 10.1016/j.foodchem.2021.129125

[fsn33790-bib-0123] Yenasew, A. , & Urga, K. (2023). Effect of the germination period on functional properties of finger millet flour and sensorial quality of porridge. Food Science and Nutrition, 11(5), 2336–2343. 37181313 10.1002/fsn3.3240 PMC10171510

[fsn33790-bib-0124] Yousaf, L. , Hou, D. , Liaqat, H. , & Shen, Q. (2021). Millet: A review of its nutritional and functional changes during processing. Food Research International, 142, 110197. 33773674 10.1016/j.foodres.2021.110197

